# Distribution and taxonomic identity of the species of *Narcine* (Toperdiniformes: Narcinidae) in the southwestern Atlantic Ocean

**DOI:** 10.1111/jfb.70106

**Published:** 2025-06-08

**Authors:** Marcus Vinicius Gonçalves Araújo, Ricardo de Souza Rosa, João Paulo Capretz Batista da Silva

**Affiliations:** ^1^ Museu de Zoologia da Universidade de São Paulo São Paulo Brazil; ^2^ Instituto de Biociências da Universidade de São Paulo São Paulo Brazil; ^3^ Laboratório de Ictiologia (LABICT), Departamento de Sistemática e Ecologia Centro de Ciências Exatas e da Natureza, Universidade Federal da Paraíba João Pessoa Brazil

**Keywords:** Batoidea, geographic distribution, numbfish

## Abstract

The genus *Narcine* is the most diverse among electric rays of the order Torpediniformes and is also the only electric ray genus with more than one species occurring in South American waters: *Narcine brasiliensis* and *Narcine bancroftii*. The first species has been historically described as distributed from Brazil's southeast coast down to northern Argentina, whereas the latter is distributed from northern South America to Central America, and the southernmost coast of North America. This revision of occurrences of the genus for the coast of Brazil, with focus on the northeast region, expands the range of distribution of both species. These species were previously considered to have very distinct areas of occurrence but actually present some degree of overlap on their distributional areas, with the northeast coast of Brazil acting as the intersection of both species' range of occurrence. Additionally, this study discusses the diagnostic characteristics for identifying *Narcine* species and highlights the historical scientific bias surrounding this group and how it ultimately can lead to a misrepresentation of its geographic distribution and conservation status.

The rays of the family Narcinidae, commonly known as numbfishes, are a group of small‐ to medium‐sized rays with a characteristically shovel‐shaped disc and a well‐developed tail, bearing two dorsal fins (Carvalho, 1999; Last, 2016). Like other Torpediniformes, the numbfishes do not have dermal denticles on their skin and are capable of generating electricity (a characteristic shared with only one other elasmobranch group, the family Rajidae) (Alves‐Gomes, 2020; Bratton & Ayers, 1987; Carvalho, 1999; Macesic & Kajiura, 2009; Moller, 1996), which sets the order apart from the other elasmobranchs. In Torpediniformes, the electric discharge is produced by a pair of bean‐shaped electric organs on their disc, which are located laterally to their branchial basket (Bigelow & Schroeder, 1953), and is mainly used for defence against predators, prey capture and intraspecific communication (Belbenoit, 1986; Belbenoit & Bauer, 1972; Bray & Hixon, 1978; Lowe et al., 1994; Macesic & Kajiura, 2009). Narcinidae is the most diverse family of Torpediniformes, with 32 currently valid species (Fricke et al., 2025) distributed almost globally (with exception of the Mediterranean Sea, the east Atlantic Ocean and Southeast Pacific Ocean) (Carvalho & Last, 2016). The representatives of the family are bottom dwellers associated with the benthos, feeding mainly on invertebrates (Carvalho & Last, 2016; Goitein et al., 1998).

Within the family Narcinidae, *Narcine* is the only genus with more than one species present along the South American coast (Carvalho, 1999; Last, 2016; McEachran et al., 2002), namely *Narcine brasiliensis* (Olfers, 1831), known as the lesser electric ray, and *Narcine bancroftii* (Griffith & Smith, 1834), commonly known as the Caribbean numbfish. These species share several morphological similarities: both species have a light sandy‐brown dorsal background colouration, sometimes grey with a distinct dark‐brown antorbital blotch, darker than the main disc colour; and a dorsal disc lacking four to five distinct ocelli with darker centres and lighter outlines in relation to the background colour; and they reach similar sizes when mature, generally ~380 mm total length (TL), although *N. bancroftii* is believed to reach greater proportions of general size (Carvalho, 1999). Nevertheless, the taxonomy of the genus is outdated, with the last systematic revision dating from the late 1990s (Carvalho, 1999), besides a possible new species reported more than 20 years ago that remains undescribed (Carvalho & McEachran, 2002).

Regarding their distribution, *N. brasiliensis*, once considered a wide‐ranging species, is now described with its distribution restricted to the southwestern Atlantic Ocean, from Espírito Santo in Brazil to the Argentinian northern coast (Carvalho, 1999; Costa & Chaves, 2007; McEachran et al., 2002; Martins et al., 2009). On the contrary, *N. bancroftii* is believed to occur from the southernmost part of the North American coast, from North Carolina, through the Gulf of Mexico and the Caribbean (Carvalho, 1999; Last, 2016), with some authors considering the northern region of the South American coast as their southernmost limit of distribution (McEachran et al., 2002; Rolim et al., 2015). This reported geographic distribution completely disregards the areas encompassing the whole northern and northeastern coast of South America. However, data from grey literature (an unpublished monograph from Araújo, 2007) and common popular knowledge indicate that the distribution of the genus should be expanded beyond what is generally reported in the literature, with probable occurrences of one or both species along the northeastern coast of Brazil, a region that has been reported by only a few authors (Last, 2016; Mathewson et al., 1958) but which is generally overlooked.

Therefore, a bibliographical survey was carried out to collect information on the South American distribution of *Narcine* documented in published literature. Additionally, specimens of *Narcine* captured along Brazil's northeast coast were examined and had their morphology analysed comparatively to southeastern specimens. We used Carvalho's identification key for the genus (1999), as it remains as the only key for species identification; 58 specimens were examined, with 30 housed at the Universidade Federal da Paraíba (UFPB) and 28 at the Museu de Zoologia da Universidade de São Paulo (MZUSP). Out of the latter 28 specimens, 3 were collected in the northeast region, whereas the rest are specimens from the southeast of Brazil used for comparisons. For morphological data, 41 different measurements were taken for each specimen following McEachran et al. (2002). The measurements were then converted into percentages of disc width. The measurements of the northeastern and southeastern specimens of *N. brasiliensis* (Figure 1) along the coast of Brazil reveal that the southeastern specimens are comparatively larger than the northeastern specimens, even for those that share similar colourations.

**FIGURE 1:**
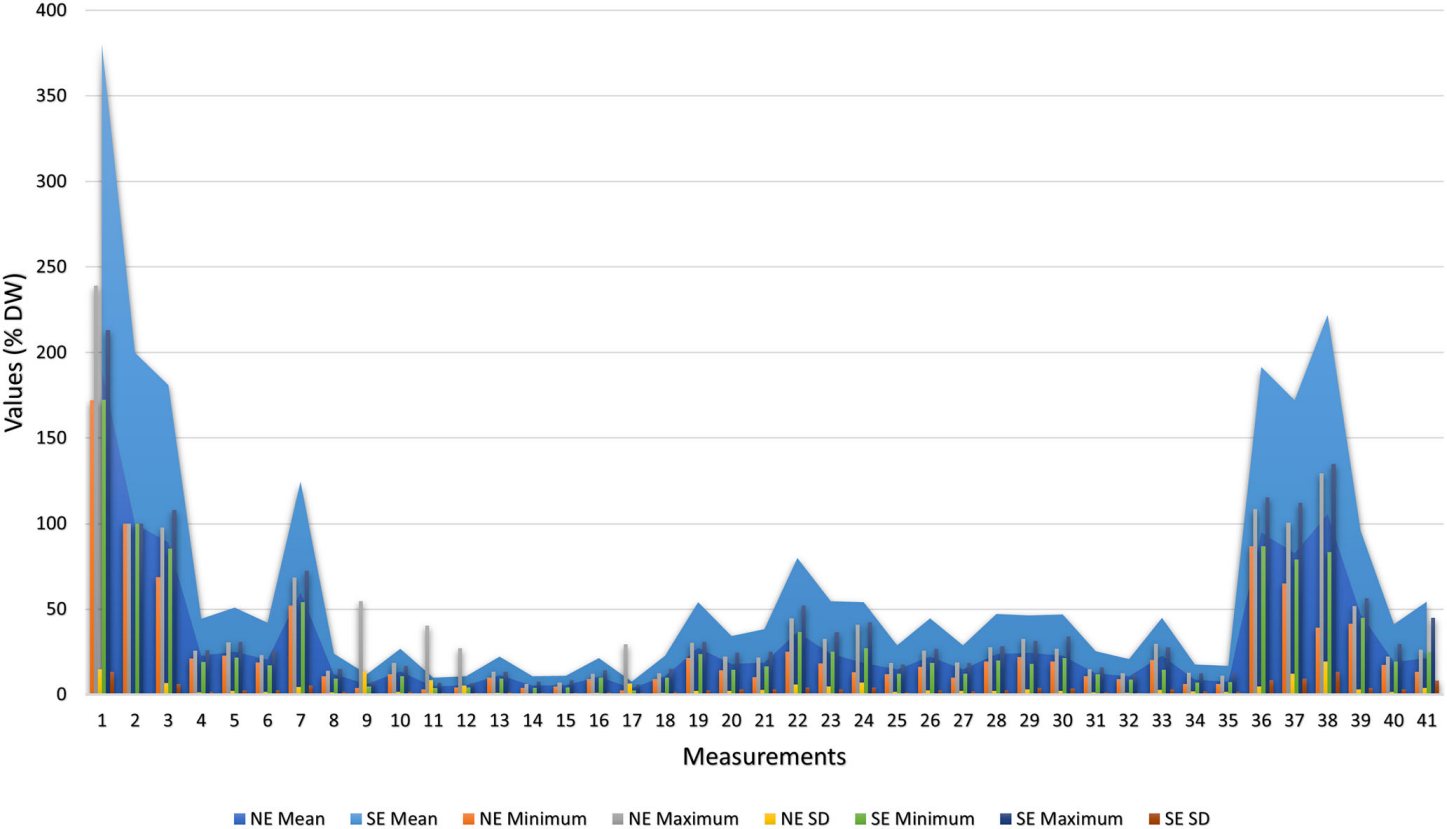
Morphometrics of the specimens of *Narcine*. NE, northeast; SE, southeast; SD, standard deviation.

Carvalho (1999) defined the colouration pattern and number of rows of exposed teeth as diagnostic characters to differentiate the two species. Based on these proposed characters, we were able to discriminate two distinct colour patterns in the analysed specimens (Figure 2). The most common colour pattern documented falls within the range of variation described for *N. brasiliensis*: dorsal colouration consisting of dark‐brown and horizontally oriented stripes over the disc and tail, with a significant dark and large blotch on its snout that extends posteriorly towards the region of the eyes (Figure 2a). The second colouration pattern was observed in a single specimen (UFPB 3048) from the municipality of Lucena, coast of Paraíba, which is closer to the description provided for the species *N. bancroftii* (Carvalho, 1999): dorsal colouration with incomplete circles and ocelli formed by a series of small dark‐brown spots along the disc and tail, with a similar snout colour pattern to *N. brasiliensis* (a significant darker and large blotch on the preocular region of disc) (Figure 2c).

**FIGURE 2:**
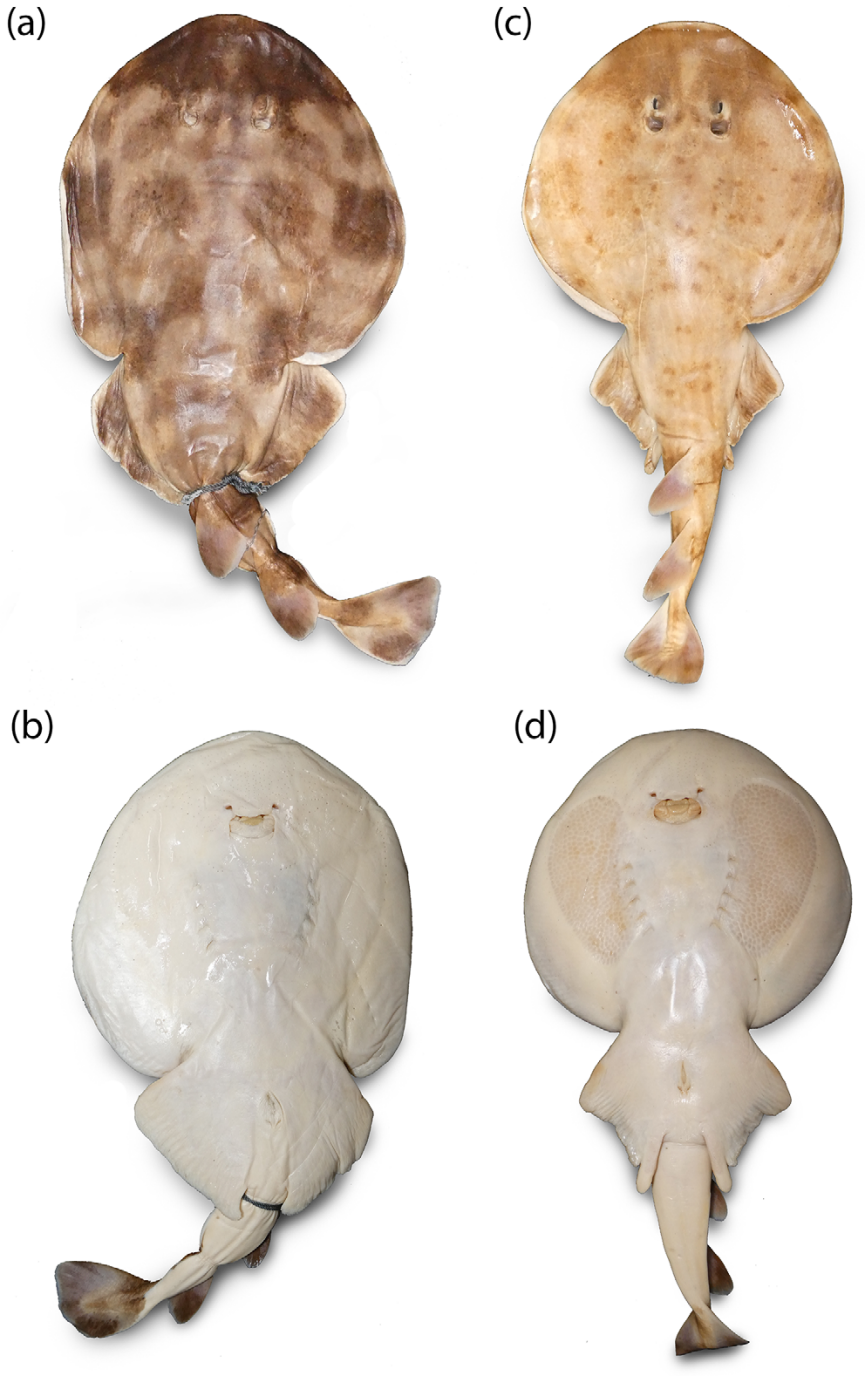
*Narcine* species colouration patterns. (a) *Narcine brasiliensis* dorsal colouration [UFPB 3055, female, 310 mm TL (total length)]; (b) *N. brasiliensis* ventral colouration; (c) *Narcine bancroftii* (UFPB 3048, male, 270 mm TL) dorsal colouration; (d) *N. bancroftii* ventral colouration.

The rows of exposed teeth, however, show a different count from what is present in the literature regarding the delimitation of these species, with a greater range of variation for specimens of *N. brasiliensis*. Carvalho (1999) indicated that the large adults would have 31/32 teeth rows (31 rows on their upper jaw and 32 on their lower jaw). Nevertheless, most of the specimens fail to reach such a count, even those large adults that reach or surpass 380 mm TL (Table 1). Using only this character for identification, those specimens would be identified as representatives of *N. bancroftii*, which is considered to have fewer rows of exposed teeth (22/23). However, their colouration overlaps the pattern observed in *N. brasiliensis*, leaving the process of species identification confusing when using the only key of identification available for the group. Another issue that may explain the difference between tooth counts is of preservational nature: the specimens examined here are not the same as those seen by Carvalho on his revision, and the method in which these two sets of specimens were handled and preserved might have influenced the process of counting what is considered exposed teeth. Although many of the aforementioned characteristics are shared between the two species, and the characters currently used for identification may contradict each other, the validity of both species is further supported by molecular data that have shown some level of delimitation between them (Gaitán‐Espitia et al., 2016). Even though some authors have pointed out to the existence of great intraspecific or ontogenetic variability in colouration in other elasmobranch species, especially in other groups of rays (Silva & De Carvalho, 2015; Silva & Silva Loboda, 2019), for the *Narcine* species analysed here the colour pattern was the character with presumably less intraspecific variation and the main one used for their identification.

**TABLE 1:** Number of exposed vertical tooth rows of species of *Narcine* in the southwestern Atlantic Ocean compiled over a period of four decades.

Catalogue number	Species	Colouration pattern	TL (mm)	Upper exposed vertical tooth rows	Lower exposed vertical tooth rows	Locality	Year	State
UFPB 3048	*Narcine bancroftii*	II	270	23	24	Lucena	1994	PB
UFPB 538	*Narcine brasiliensis*	I	493	23	21	Rio Paraíba, Costinha, Lucena	1980	PB
UFPB 771	*N. brasiliensis*	I	308	22	23	Praia da Costinha, Lucena	1981	PB
UFPB 1471	*N. brasiliensis*	I	313	21	22	Praia do Forte, Cabedelo	1980	PB
UFPB 2667	*N. brasiliensis*	I	247	18	19	Praia de Tambaú	1993	PB
UFPB 2734	*N. brasiliensis*	I	290	22	22	Praia de Jacumã	1993	PB
UFPB 2735	*N. brasiliensis*	I	460	23	20	Praia de Jacumã, Conde	1993	PB
UFPB 2739	*N. brasiliensis*	I	350	23	22	Praia de Jacumã	1993	PB
UFPB 2739'	*N. brasiliensis*	I	280	24	26	Praia de Jacumã	1993	PB
UFPB 2739″	*N. brasiliensis*	I	258	21	21	Praia de Jacumã	1993	PB
UFPB 3051	*N. brasiliensis*	I	357	26	24	Lucena	1995	PB
UFPB 3051'	*N. brasiliensis*	I	460	23	23	Lucena	1995	PB
UFPB 3252	*N. brasiliensis*	I	302	23	24	Lucena	1995	PB
UFPB 3255	*N. brasiliensis*	I	442	23	20	Lucena	1995	PB
UFPB 3255'	*N. brasiliensis*	I	310	20	21	Lucena	1995	PB
UFPB 3823	*N. brasiliensis*	I	390	25	23	Rio Mamanguape, Barra de Mamanguape	1998	PB
UFPB 5987	*N. brasiliensis*	I	280	23	23	Praia do Amor, Conde	2004	PB
MZUSP 51829	*N. brasiliensis*	I	250	18	17	Near Rio Doce	1969	ES
MZUSP 51833	*N. brasiliensis*	I	245	16	15	–	1972	RS
MZUSP 51833'	*N. brasiliensis*	I	232	17	16	–	1972	RS
MZUSP 72784	*N. brasiliensis*	I	255	21	22	Praia de Itaguá, Ubatuba	1970	SP
MZUSP 72793	*N. brasiliensis*	I	272	19	17	–	1970	–
MZUSP 72795	*N. brasiliensis*	I	258	17	17	–	1972	RS
MZUSP 72796	*N. brasiliensis*	I	265	22	22	Cananéia	1971	SP
MZUSP 72800	*N. brasiliensis*	I	300	20	21	Ubatuba	1971	SP
MZUSP 72800'	*N. brasiliensis*	I	359	25	26	Ubatuba	1971	SP
MZUSP 116353	*N. brasiliensis*	I	265	20	19	–	–	–
MZUSP 51830	*N. brasiliensis*	–	–	–	–	Rio Curuçá, São Luís	1983	MA
MZUSP 51832	*N. brasiliensis*	–	–	–	–	Aracaju	1961	SE
MZUSP 79876	*N. brasiliensis*	I	193	19	21	Praia da Avenida, Maceió	1978	AL
MZUSP 52987	*N. brasiliensis*	I	97	13	12	Ilha Siriba, Arquipélago de Abrolhos	1997	BA
Uncat.	*Narcine* cf. *brasiliensis*	–	–	–	–	Praia do Porto da Barra, Salvador	2008	BA
Uncat.	*Narcine* cf. *brasiliensis*	–	–	–	–	Baía de todos os santos, Salvador	2013	BA
Uncat.	*Narcine* cf. *brasiliensis*	–	–	–	–	Prainha, Cairu	2019	BA
Uncat.	*Narcine* cf. *brasiliensis*	–	–	–	–	Arquipélago de Abrolhos	2022	BA

Abbreviations: I, colour pattern of *N. brasiliensis* described by Carvalho (1999); II, colour pattern of *N. bancroftii* described by Carvalho (1999); AL, Alagoas; BA, Bahia; ES, Espírito Santo; MA, Maranhão; PB, Paraíba; RS, Rio Grande do Sul; SE, Sergipe; SP, São Paulo; TL, total length (mm); Uncat., records of specimens that are not housed in any ichthyological collection and are available at iNaturalist.

Of the 34 specimens captured along the northeastern Brazilian coast, 33 were identified as *N. brasiliensis*. Of these, 17 were mature individuals (9 females and 8 males), whereas 17 were juveniles. As previously mentioned, a single specimen was identified here as *N. bancroftii*, a juvenile male (270 mm TL). The majority of the specimens were collected along the coast of the state of Paraíba (28 specimens), with the remaining individuals originating from four other states of the same region (e.g. Alagoas, Maranhão, Sergipe and Bahia) (Table 1). Additionally, four records found on the website iNaturalist were included in our analysis, with records of sightings of species of *Narcine* on the coast of the state of Bahia (Figure 3), with 37 records of *Narcine* for this region of Brazil. Out of the specimens observed here, five were presumably captured in brackish‐water environment: MZUSP 051830, captured along the Rio Curuçá, in the São Luís island, MA; UFPB 538, captured in Rio Paraíba, Costinha, in Lucena, PB; UFPB 3823, captured in Rio Mamanguape, Rio Tinto, PB; MZUSP 4821, Rio Aguapéu, Itanhaém, SP; and MZUSP 9734, Rio Paraíba, São João da Barra, RJ. It is worth mentioning that all Torpediniformes, including the numbfishes, are exclusively marine fishes. The record of species of *Narcine* in river mouths most likely reflects a tolerance in relation to the variation in water salinity, as opposed to the ability to completely inhabit such an environment.

**FIGURE 3:**
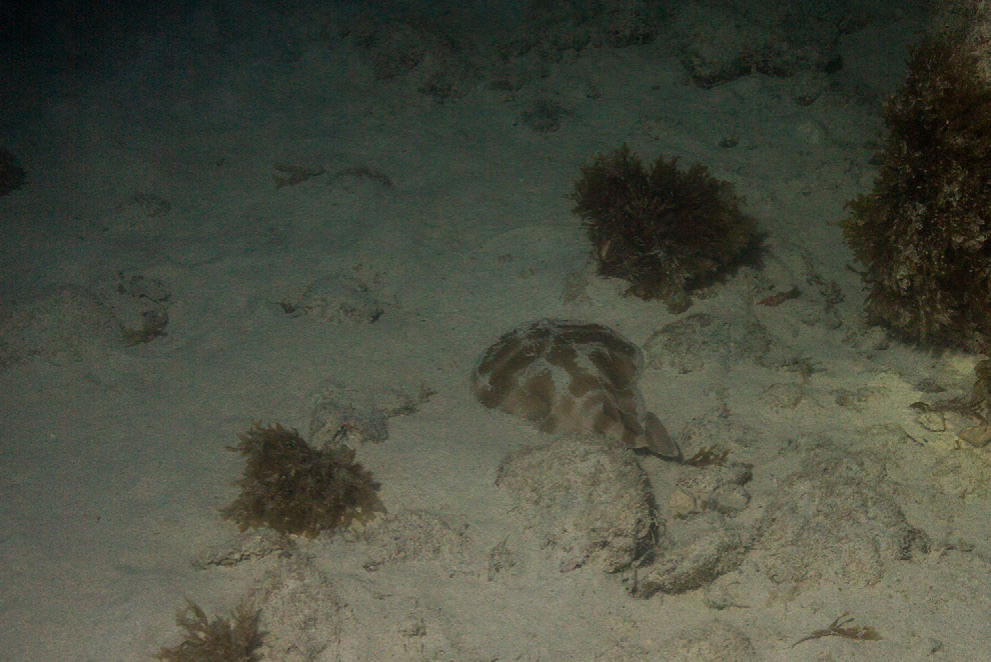
Sighting of *Narcine* specimen in the Abrolhos archipelago, BA, 2022. Image credits: João D'Andretta. *Source*: https://www.inaturalist.org/observations/137429836 .

Records of numbfishes for the northeast coast of Brazil are not new: the earliest record in the present study dates back to a specimen collected in 1961 in the state of Sergipe, followed by records of specimens being regularly captured throughout the 1980s, 1990s and late 2000s (Gadig, 1993; Gadig et al., 2000; Guedes et al., 1989; Lessa, 1986; Menni & Lessa, 1998; Menni & Stehmann, 2000; Queiroz et al., 1993) (Table 1). On the coast of Paraíba, where most of the specimens of the present study came from, the distribution of the genus covers practically the entire coast (Figure 4). However, the northeast coast of Brazil was not mentioned as a possible region of occurrence in the last revision of the genus made by Carvalho (1999). As a consequence, the vast majority of the subsequent published data regarding Narcine in Brazil comes from the country's southeast region, where a greater effort in capture as well as scientific endeavours has been made (Costa & Chaves, 2007; Ferreira & Vooren, 2012; McEachran et al., 2002; Rolim et al., 2015, 2016; Rolim, Rotundo, et al., 2020; Rolim, Siders, et al., 2020; Vianna & Vooren, 2009). This resulted in an historical geographic bias in the literature of the group, with its northern and northeastern distribution being still poorly documented and almost entirely neglected, leading to an incomplete and imprecise understanding of its distribution in the southwestern Atlantic Ocean. This gap in the distribution of *Narcine* can also be seen in the IUCN's (International Union for the Conservation of Nature) conservation status of both species, where *N. bancroftii* and *N. brasiliensis* are still considered absent from the northeastern coast of South America (Driggers & Carlson, 2019; Pollom et al., 2020). Only a limited number of authors (Araújo et al., 2022; Dean et al., 2005; Dean & Motta, 2004a; Dean & Motta, 2004b; Last, 2016; Mathewson et al., 1958) recognized the existence of *N. brasiliensis* beyond the northern limit described by Carvalho (1999). For instance, Last (2016) argued that *N. brasiliensis* would have a wider range and would be found along almost the entire coast of Brazil. Our data are in accordance with these previous works, with *N. brasiliensis* ranging far north beyond the currently established limit of distribution defined for the species.

**FIGURE 4:**
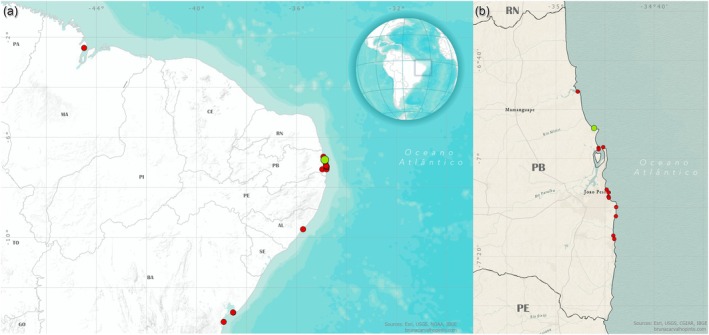
*Narcine* distribution in northeastern South America. (a) Records of *Narcine* species along the northeastern coast of Brazil. (b) Records of *Narcine* throughout the coast of Paraíba state. Red circles, records of *Narcine brasiliensis*; green circles, records of *Narcine bancroftii*.

This study highlights the gap present in the literature regarding the genus Narcine and further supports its formerly wider distributional range as defended by Mathewson et al. (1958), Dean and Motta (2004a), Dean and Motta (2004b) and Last (2016). It is also clear that the morphological characters generally used to identify the *Narcine* species exhibit relative variation and overlap, making them unsuitable and even unreliable as the only source for species delimitation. Such poor understanding of the taxonomic limits and distribution of these species, along with the existing regional bias in the production of scientific knowledge, may have direct impacts on the conservation of this group. Thus, we emphasize the need for future studies, especially a thorough and urgent revisionary study of the genus in its Atlantic range of occurrence, given the fact that both South American species bear a striking resemblance. Only with an approach like this will the conservation status of these species closely reflect their reality in their natural habitat.

## AUTHOR CONTRIBUTIONS

Marcus Vinicius Gonçalves Araújo: conceptualization, data collection, manuscript writing and editing. João Paulo Capretz Batista da Silva: project supervision and manuscript editing. Ricardo de Souza Rosa: project supervision.
